# Analysis and Design Methodology of RF Energy Harvesting Rectifier Circuit for Ultra-Low Power Applications

**DOI:** 10.1109/ojcas.2022.3169437

**Published:** 2022-04-21

**Authors:** ZIYUE XU, ADAM KHALIFA, ANKIT MITTAL, MEHDI NASROLLAHPOURMOTLAGHZANJANI, RALPH ETIENNE-CUMMINGS, NIAN XIANG SUN, SYDNEY S. CASH, AATMESH SHRIVASTAVA

**Affiliations:** 1 Department of Electrical and Computer Engineering, Northeastern University, Boston, MA 02115, USA; 2 Department of Neurology, Massachusetts General Hospital and Harvard Medical School, Boston, MA 02114, USA; 3 Department of Electrical and Computer Engineering, Johns Hopkins University, Baltimore, MD 21218, USA

**Keywords:** RF energy harvesting, RF rectifier, Internet-of-Things (IoT), implantable device, matching network, ultra-low-power

## Abstract

This paper reviews and analyses the design of popular radio frequency energy harvesting systems and proposes a method to qualitatively and quantitatively analyze their circuit architectures using new square-wave approximation method. This approach helps in simplifying design analysis. Using this analysis, we can establish no load output voltage characteristics, upper limit on rectifier efficiency, and maximum power characteristics of a rectifier. This paper will help guide the design of RF energy harvesting rectifier circuits for radio frequency identification (RFIDs), the Internet of Things (IoTs), wearable, and implantable medical device applications. Different application scenarios are explained in the context of design challenges, and corresponding design considerations are discussed in order to evaluate their performance. The pros and cons of different rectifier topologies are also investigated. In addition to presenting the popular rectifier topologies, new measurement results of these energy harvester topologies, fabricated in 65nm, 130nm and 180nm CMOS technologies are also presented.

## I. INTRODUCTION

Energy harvesting from solar, thermal, vibration, and radio-frequency (RF) are increasingly being used to realize batteryless operation for Internet-of-Things (IoT) and biomedical applications [1]–[4]. Among these techniques, RF energy harvesting is particularly promising for biomedical devices where other sources are not readily available. RF energy harvesting is also employed for the RF identification (RFID) system, in IoT applications, among others. Several of these applications are utilizing widely used WiFi and Bluetooth low-energy (BLE) communication standards. These applications along with the wirelessly-powered neural implantable medical devices (*n*-IMD) for neural stimulation and recording are also benefiting from ultra-low power (ULP) circuits and systems design advancements [1].

For RF energy harvesting applications, integrated circuit (IC) design goals include developing high efficiency energy harvesting circuits across a wide input power range to maximize the output power. Often, we also need to maintain a minimum required input power level to maintain the normal functionality of the system. These circuits and system design approaches have provided new design methods as well as analysis for underlying circuits designs [5]–[7]. Most applications mentioned above utilize the characteristics of RF-to-DC rectifier but often also need to address concerns in their application space. Several applications, including *n*-IMD require higher level of on-chip integration to keep the device size small for lower cost, decreased invasiveness, and higher longevity [8]–[10]. As the size of the energy harvester along with the matching network decreases, the optimal powering frequency increases [11] which drives the operating frequency in the sub-GHz to tens of GHz range [8].

**FIGURE 1.**
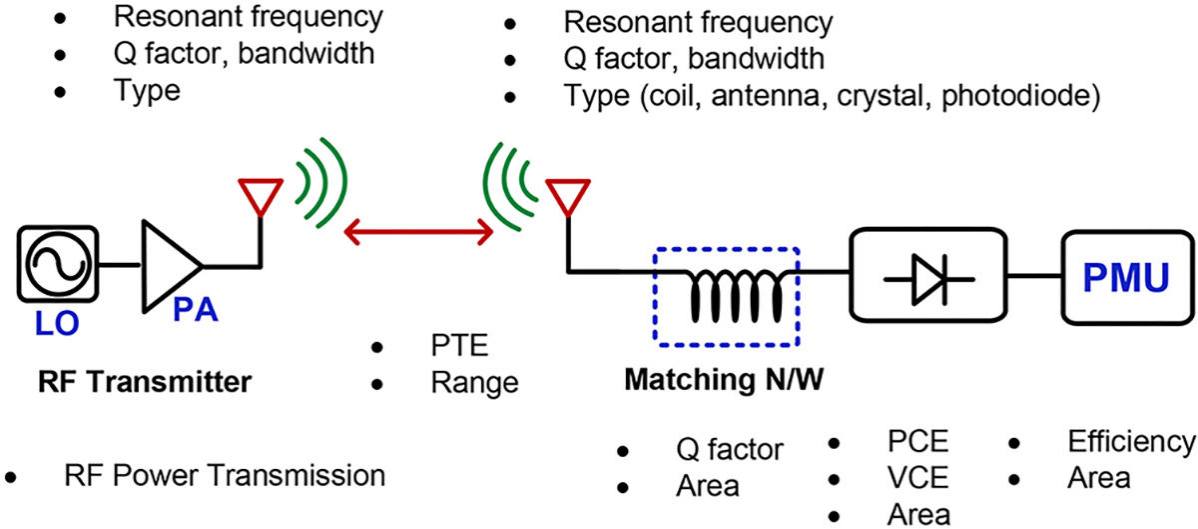
Block diagram of a typical radio frequency energy harvesting system. Design parameters that need to be optimized are shown for each stage.

A typical RF energy harvesting system configuration is shown in Fig. 1. The antenna receives the RF signal and transmits it to the rectifier through a matching network. The rectifier converts the RF into a DC output. The main challenges of this procedure is to achieve a high power conversion efficiency (PCE) which is defined as the ratio of the delivered output power and the received input power. In most cases when the input RF signal amplitude is small, a low-loss rectifier is required in addition to a good matching network. Apart from PCE, the other important parameters such as sensitivity and output DC voltage can also be used to evaluate the system performance. The sensitivity means the minimum input power level that can achieve the desirable output voltage.

While several circuit architectures are generally well known [7], [12]–[15], the analysis of underlying circuits is quite involved. In [6], [16], [17], authors have performed analysis to obtain the model for output voltage and power of a rectifier. However due to complex equations and dependence of output on several design parameters, design optimization task is not simplified. In this paper, we propose a new square-wave approximation to achieve a simpler analysis. This helps us in obtaining design insights and upper limits on circuit performance which has not been reported previously. The main contributions of this paper are; (i) a new simplified method for analyzing RF rectifier topologies using the square-wave approximation, (ii) design optimization techniques based on the simplified analysis, and (iii) measurement results across three technologies to validate the analysis and optimization method.

Using the proposed analysis, we can establish (i) independence of no-load output voltage of rectifiers on its threshold voltage, (ii) upper limit on its efficiency, and (iii) its output power characteristics. We draw these conclusions using square-wave approximation which cannot be easily drawn from analysis carried out in prior works. Further, we present a comparative study of designs across technologies through chip measurement results which will also provide useful perspective for designers. The paper is organized as following. Our analysis is presented in Section II. Section III presents other topology of energy harvesters. Section IV discusses how different applications utilize the physical mechanism of the energy harvester to serve their purposes and other design considerations, e.g., matching network design and the EMI effect. The discussion in the paper will be substantiated by several measurement results in Section V, including chip measurement results where new unpublished measurement data and circuit designs are also be included.

## II. BASIC RECTIFIER ARCHITECTURE

**FIGURE 2.**
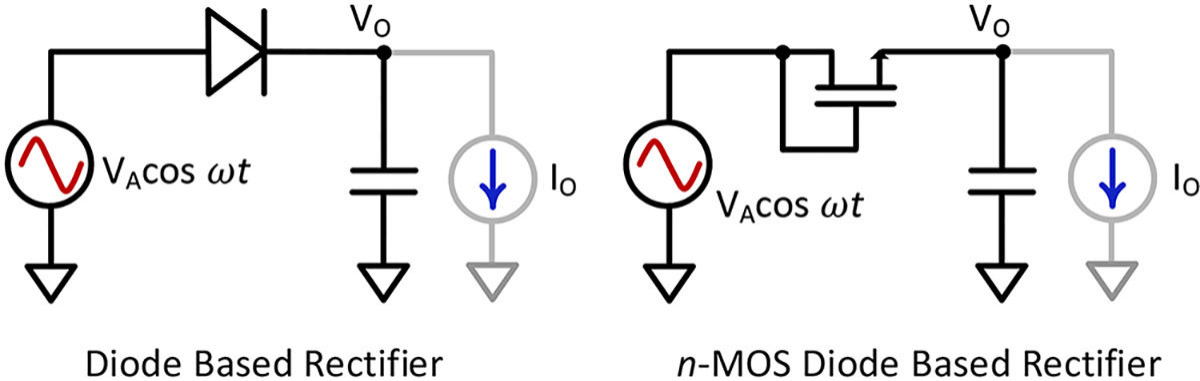
Half-wave diode rectification circuits.

RF energy harvesting circuits are built from multiple stages of a rectifier circuit. A typical half-wave rectifier is composed of a diode and a capacitor as shown in Fig. 2. In IC design, the rectifier stage is typically implemented using transistors as a diode as shown in Fig. 2. The input power received in RF energy harvesting applications is often low [18] due to FCC regulations on output power from a transmitter and associated path loss. Transistors operated as a diode in RF energy harvester circuits therefore operate more often in the sub-threshold or near-threshold regions. The usual RF input power is lower than −10dBm, even in cases where transmitter is very close to the receiver [5], [13], [19]. For this analysis, we will primarily focus on sub-threshold and near-threshold operation of the transistor.

### A. HALF-WAVE RECTIFIER DESIGN

The half-wave rectifier stage as shown in Fig. 2 will provide an output voltage *V*_*O*_ and an output current *I*_*O*_. For a fixed output load, *V*_*O*_ will be close to a DC voltage with small ripple as rectifier capacitor is usually large. The drain current of a transistor operated in the sub-threshold region is given by

(1) $$I_{D}=I_{S}e^{\left(V_{GS}-V_{TH}\right)/\eta V_{t}}\left(1-e^{-V_{DS}/V_{t}}\right)$$

where *I*_*S*_ is the sub-threshold saturation current, *η* is the ideality factor, and *V*_*t*_ is the thermal voltage given by *kT*/*q*. For a diode in the RF energy harvester, its *V*_*GS*_ = *V*_*O*_ − *V*_*A*_*cosωt*. The average current from the rectifier can be calculated by

(2) $$I_{O}=\frac{1}{T}\cdot \int_{0}^{T}I_{D}\cdot dt$$

**FIGURE 3.**
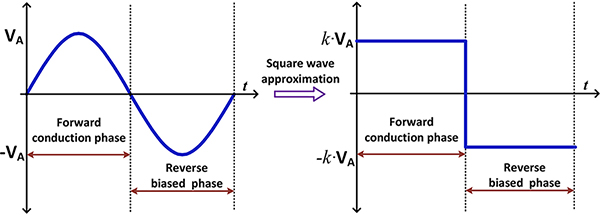
Square wave approximation for analysis.

However, this results in an involved output equation [20]. To further simplify the analysis, we approximate the sinusoidal input of an amplitude *V*_*A*_ with a square wave with an approximate amplitude of *k* · *V*_*A*_ as shown in Fig. 3. We assume that the scaling factor *k* for the square wave is such that both square wave and the sine wave input provides same output power. We will later show that this approximation is valid and provides a simplified solution for the output current, voltage, and power of the rectifier circuit. The resulting equation assuming a square wave input is given by

(3) $$I_{O}≅\frac{I_{ST}}{T}\cdot \int_{0}^{T/2}e^{\left(k\cdot V_{A}-V_{O}\right)/\eta V_{t}}\left(1-e^{\left(-k\cdot V_{A}+V_{O}\right)/V_{t}}\right)\cdot dt\;\;-\frac{I_{ST}}{T}\cdot \int_{T/2}^{T}e^{(0)/\eta V_{t}}\left(1-e^{-\left(k\cdot V_{A}+V_{O}\right)/V_{t}}\right)\cdot dt$$

where $I_{ST}=I_{S}\cdot e^{-V_{TH}/\eta V_{t}}$, and *V*_*GS*_ = 0 during the reversed bias phase. The equation resolves to

(4) $$I_{O}=\frac{I_{ST}}{2}\left[e^{\left(k\cdot V_{A}-V_{O}\right)/\eta V_{t}}\left(1-e^{\left(-k\cdot V_{A}+V_{O}\right)/V_{t}}\right)-1\right]$$

The expression of *I*_*O*_ is the general expression that can be further analyzed for two popular energy harvesting scenarios. In one scenario, we focus on the maximum efficiency of the energy harvesting system. In another scenario, no-load output voltage (*V*_*ON*_) is of interest as it provides the maximum energy storage for the system through $E=C_{OUT}V_{O}^{2}/2$.

#### No-load Output Voltage:

The no-load output voltage is given by eq. (4) when *I*_*O*_ = 0 and results into the following solution.

(5) $$\left[e^{\left(k\cdot V_{A}-V_{ON}\right)/\eta V_{t}}\left(1-e^{\left(-k\cdot V_{A}+V_{ON}\right)/V_{t}}\right)-1\right]=0$$

(6) $$\left[\left(1-e^{\left(-k\cdot V_{A}+V_{ON}\right)/V_{t}}\right)-e^{\left(-k\cdot V_{A}+V_{ON}\right)/\eta V_{t}}\right]=0$$

Assuming that *V*_*ON*_ and *k* · *V*_*A*_ are close, we obtain,

$$\left[\left(1-\left(1-\frac{-k\cdot V_{A}+V_{ON}}{V_{t}}\right)\right)-\left(1-\frac{-k\cdot V_{A}+V_{ON}}{\eta V_{t}}\right)\right]=0$$

This results in the output expression for *V*_*ON*_ as

(7) $$V_{ON}=k\cdot V_{A}-V_{t}\left[\frac{\eta }{1+\eta }\right]$$

Several conclusions can be drawn from eq. (7) that shed light on the rectifier design. First, it shows that the only process dependent parameter that affects *V*_*ON*_ is *η* indicating that transistor choice and size has a very low impact on the value of *V*_*ON*_ reached. Second, *V*_*ON*_ is a linear function of *V*_*A*_ which is anticipated because output should increase with input amplitude. Finally, it also shows *V*_*ON*_ to be complimentary to absolute temperature (CTAT) due to the −*V*_*t*_ term. Another useful conclusion is that the rectifier will not produce meaningful output voltage for *V*_*A*_ ⪅ *V*_*t*_ as eq. (7) shows that *V*_*ON*_ will be negative in that case.

A key outcome of eq. (7) is independence of output voltage on *V*_*TH*_ which can be explained qualitatively as well. The average output current is zero in the no load condition which means that the sum of currents in the forward conduction path is equal to the leakage current in the reverse conduction path. In the reverse conduction path, *V*_*GS*_ = 0 and therefore the leakage is dependent on *V*_*TH*_. In the forward conduction path, *V*_*GS*_ will only be slightly higher than 0 and forward current is also determined by *V*_*TH*_. This means that a low *V*_*TH*_ transistor, which can supply high current in the forward path, will also have high leakage in the reverse path. Similarly a high *V*_*TH*_ transistor will have low conductance in the forward path but since its leakage is low as well, it will still reach higher voltage. Since currents in the forward and reverse phase depends on *V*_*TH*_ in the same way, therefore no load output shows no dependence on *V*_*TH*_ in the first order. The charging and discharging currents are influenced in the same way by *V*_*TH*_.

**FIGURE 4.**
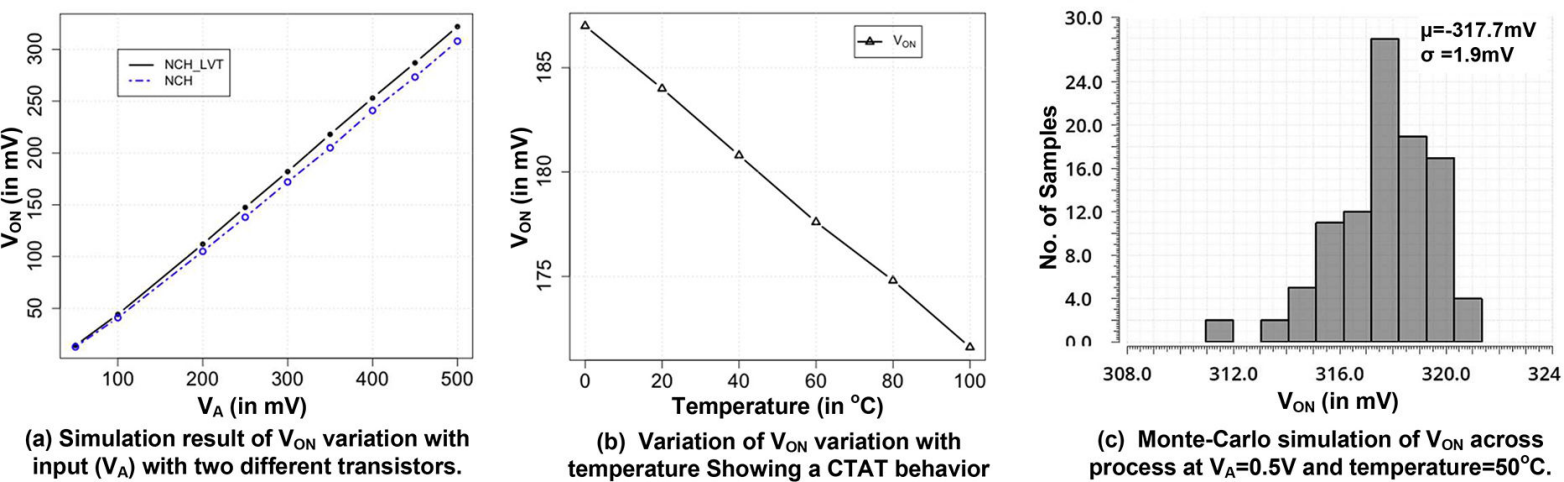
Simulation results of a half-wave rectifier stage output in no-load case.

To verify the results obtained from eq. (7), we performed the simulation of a single-stage rectifier across various conditions and with different transistor types. The output capacitors used in these simulations were very large based on energy harvesting applications. Simulations were carried out using foundry supplied process design kit (PDK) which uses comprehensive BSIM-4 model of transistors in 65nm CMOS technology. We performed the SPICE simulation of the circuit based on these models using Cadence Spectre SPICE simulation tool. Fig. 4a shows the simulation result of *V*_*ON*_ with varying *V*_*A*_. We used an RF signal at 1.7*GHz* as *V*_*A*_ for this simulation. Two different transistors, NCH (*V*_*TH*_=0.48V) and NCH_LVT_ (*V*_*TH*_=0.3V) in 65nm CMOS technology were used for these simulations. Despite a 180*mV* difference in their threshold voltage, both designs produce almost the same output voltage. A difference of 14*mV* when *V*_*ON*_ is 322*mV* is seen when *V*_*A*_ is 0.5*V*. We also note that the output remains linear with varying input power. This is an important outcome considering the square-wave approximation. We would see a non-linear *V*_*ON*_ behavior if *k* was not a constant and changing with input voltage. However, a linear *V*_*ON*_ confirms the trend predicted by eq. (7). The simulation result also indicate that the scaling factor *k* remains constant and our square wave assumption holds true across input power. This can be partly explained by the fact that the square wave contains higher order harmonics but due to low-pass nature of diode and capacitor, higher order harmonics do not contribute meaningfully to the output. Consequently, a sine wave and an equivalent square wave will show similar output voltages. Fig. 4b shows temperature variation of the single-stage rectifier output when *V*_*A*_ is 0.3*V* showing a CTAT behavior. Fig. 4c shows the simulation result of the variation of *V*_*ON*_ due to the statistical process variation. The Monte-Carlo simulation shows a 3*σ* process variation of 6% showing a low process variation while still operating in the sub-threshold region. Simulation results show that *k* has a value of 0.87.

#### Loaded Output Condition:

In the loaded output condition, the average output current, *I*_*O*_ is approximately given by eq. (4). Also, as the output current will be much larger in the forward biased case than in the reverse biased case. The leakage current in the reverse biased case can be ignored. Also, *k* · *V*_*A*_ − *V*_*O*_ >> *V*_*t*_ in loaded condition for reasonable input voltages, the output current from eq. (4) can be approximated as,

(8) $$I_{O}=\frac{I_{ST}}{2}\left[e^{\left(k\cdot V_{A}-V_{O}\right)/\eta V_{t}}-1\right]$$

The output power from the rectifier can be then written as

(9) $$P_{O}=V_{O}\cdot I_{O}=V_{O}\cdot \frac{I_{ST}}{2}\left[e^{\left(k\cdot V_{A}-V_{O}\right)/\eta V_{t}}-1\right]$$

*P*_*O*_ comes out as a function of *V*_*O*_ and a quick inspection of eq. (9) shows that when *V*_*O*_ increases *I*_*O*_ decreases. It leads to a unique value of *V*_*O*_ at which *P*_*O*_ can be maximized when *dP*_*O*_/*dV*_*O*_ = 0. For sub-threshold operation, the output power is maximized for a *V*_*O*_ value of *V*_*O*,*max*_ is given by the analytical equation,

(10) $$V_{O,max}=\eta \cdot V_{t}\left[1-e^{-\left(k\cdot V_{A}-V_{O,max}\right)/\eta V_{t}}\right]$$

Although approximate, eq. (10) indicates that the maximum output power is realized when output voltage reaches a constant voltage of close to *ηV*_*t*_. In reality, *V*_*O*,*max*_ has a weak dependence on *V*_*A*_ in sub-threshold. It also shows that the transistor type has less impact on *V*_*O*,*max*_ value in sub-threshold. It shows that as *V*_*A*_ increases, *V*_*O*,*max*_ becomes constant. However, as *V*_*A*_ increases the transistor leaves sub-threshold and goes into saturation, *V*_*O*,*max*_ is given by,

(11) $$V_{O,max}=\frac{k\cdot V_{A}-V_{TH}}{3}$$

which shows dependence on both *V*_*A*_ and *V*_*TH*_.

#### Rectifier Efficiency:

Using the square wave approximation and *V*_*O*,*max*_ values obtained from the previous analysis, we can obtain the peak efficiency expression for the rectifier. For the half wave design, the rectifier conducts only in the positive cycle while there is small leakage in the negative. Since the leakage power will be significantly smaller than the power transferred in the positive cycle, the input power can be given as,

(12) $$P_{IN}=k\cdot V_{A}\cdot I_{IN}$$

Since rectifier conducts in only half cycle, average transferred power from the source is *k*·*V*_*A*_·*I*_*IN*_/2. Further, applying ideal impedance matching for the rectifier in the conduction cycle, maximum available power for the rectifier is 1/2 · *k* · *V*_*A*_ · *I*_*IN*_/2. Total charge transfer in one cycle using square wave approximation can be given as *I*_*IN*_ ·*T*/2 which should be equal to the total output charge of *I*_*O*_*T* which sets *I*_*IN*_ = 2*I*_*O*_. The corresponding power conversion efficiency (PCE) is given by

(13) $$PCE=\frac{P_{O}}{P_{IN}}=\frac{V_{O}I_{O}}{0.5kV_{A}I_{IN}/2}=\frac{V_{O}I_{O}}{kV_{A}I_{O}/2}=\frac{2V_{O}}{kV_{A}}$$

Using this, we obtain the expression for maximum efficiency in sub-threshold for higher values of *V_A_* can be approximately given as,

(14) $$PCE_{Sub}=\frac{2\eta V_{t}}{kV_{A}}$$

Eq. (14) shows that the maximum efficiency can be reached when *kV*_*A*_ = 2*ηV*_*t*_. Since *V*_*O*,*max*_ is close to *ηV*_*t*_ in sub-threshold, the above condition indicates impedance matching between input and output. Also, it shows that the efficiency will degrade for higher input power. This happens due to the increase in input power but output voltage remains (≈ *ηV*_*t*_) low for maximum efficiency. This means that we need to add more stages to increase the efficiency. This will increase the value *V*_*O*,*max*_ to increase the output power.

The expression for efficiency in saturation region (*kV*_*A*_ > *V*_*TH*_) using eq. (11) and (13) can be given as,

(15) $$PCE_{Sat}=\frac{2}{3}\left(1-\frac{V_{TH}}{kV_{A}}\right)$$

From this equation, we observe that rectifier efficiency can be increased by increasing the input power. We also observe that when the output power is maximum, the overall rectifier efficiency is always lower than 66.7%. This upper limit is based on approximation and slightly higher efficiencies can be realized. However, this observation is generally in line with the reported efficiencies in the literature where peak efficiency of CMOS rectifiers has remained below 70%.

**FIGURE 5.**
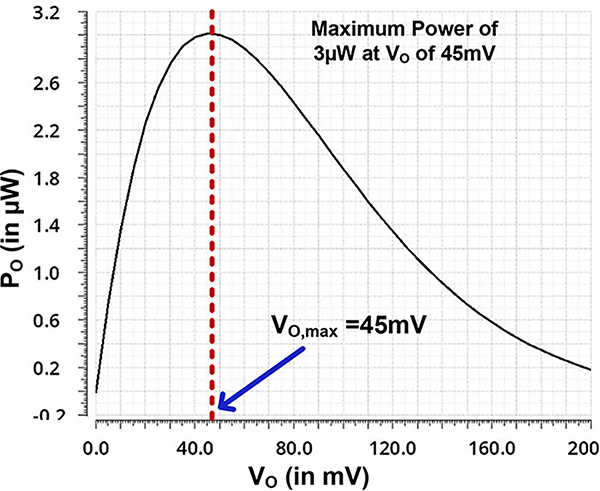
Simulation of a output power of a half-wave rectifier stage as function of output voltage with *V*_*A*_ = 0.4*V* and temperature = 27°*C.*

**FIGURE 6.**
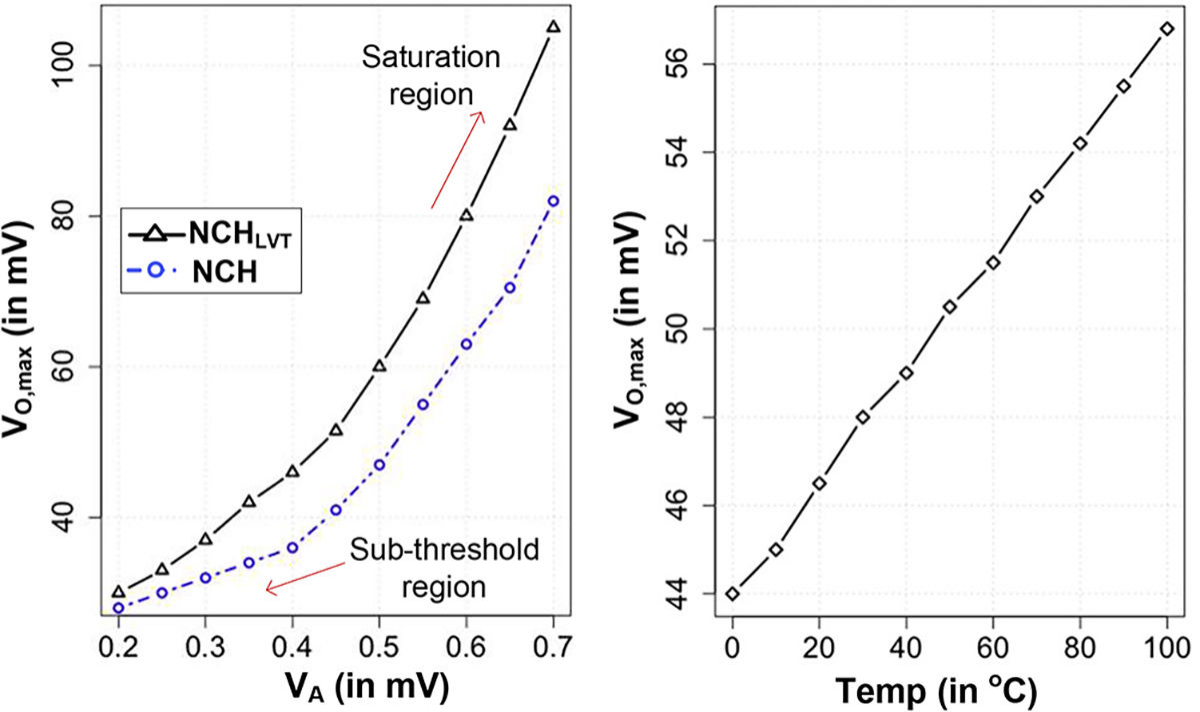
Simulation of *V_O,max_* as a function of *V*_*A*_ and temperature.

We simulated the half-wave rectifier circuit in SPICE using foundry supplied PDK model to verify our analysis. Fig. 5 shows the output power as a function of output voltage. A maximum power of 3*μW* is realized at *V*_*O*,*max*_ = 45*mV* for an input voltage of 400*mV* at 27°*C*. The output power will increase linearly for lower values of *V*_*O*_ owing to the small contribution $e^{-V_{O}/\eta V_{t}}$ function but will decrease exponentially at higher values *V*_*O*_ owing to exponential decay function due to $e^{-V_{O}/\eta V_{t}}$. In between it will have a peak. The simulation result in Fig. 5 matches closely with the trend predicted by eq. (9) verifying our underlying analysis. We further swept the *V*_*A*_ to obtain the corresponding *V*_*O*,*max*_ value. Fig. 6 shows the variation of *V*_*O*,*max*_ with *V*_*A*_ for NCH and NCH_LVT_ designs. In the sub-threshold region, the slope of the curve is small. Also, difference of *V*_*O*,*max*_ of NCH and NCH_LVT_ is small. In the saturation region, the slope of the curve is large. Note that the output power does depend on the transistor type. For maximum output power, the transistor that provides the highest current should be used. It indicates an advantage for bigger and lower *V*_*TH*_ designs. For maximum voltage designs, as discussed earlier, transistor size or types have less impact.

### B. MULTISTAGE RECTIFIER DESIGN

**FIGURE 7.**
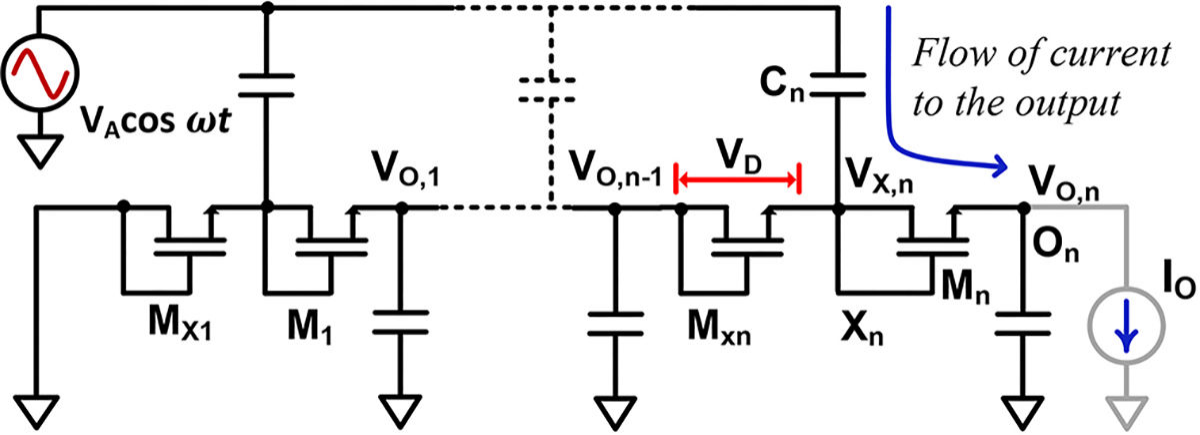
A multi-stage rectifier topology.

The half-wave rectifier analysis carried out in the previous section lays the foundation for analyzing multi-stage (*n*-stage) designs. Fig. 7 shows the circuit architecture of a multi-stage rectifier we analyze in the following section.

#### Operating Principle:

The multi-stage rectifier operates in the following manner. When the RF input is negative, *M*_*X*1_ is forward-biased and *M_1_* is reverse biased. The DC node 1 voltage (*V_1_*) is slowly charged up whenever *M_1_* is conducting through the forward bias. During the positive cycle, *M_1_* is conducting when the voltage difference between *V_1_* and *V*_*O*,1_ is positive or higher than the threshold voltage and the charge accumulated during the previous cycle on *C_1_* is dumped to *C_2_* until there is no voltage difference. By stacking multiple number of stages (*n*), the output voltage can be built up to the desirable level according to the application requirements.

#### No-load Output Voltage:

The no-load output voltage of a half-wave rectifier can be directly applied in a multi-stage rectifier. The no-load output voltage of the first half-wave stage, in a multi-stage rectifier, will be the same as the output of a stand-alone half-wave rectifier. For the next stage, its input voltage swings on top of the DC level of the first stage, which will result in an output voltage 2× of the half-wave rectifier and so on. Note that the body biasing of the transistor will result in an elevated threshold voltage of the higher stage transistors. However, our analysis for the single stage design reveals that no-load output voltage does not depend on the threshold voltage which will also apply here. Finally, the no-load output voltage for an *n*-stage rectifier, *V*_*O*,*n*_ is given by

(16) $$V_{O,n}=2n\cdot V_{ON}≅2n\left\{k\cdot V_{A}-V_{t}\left[\frac{\eta }{1+\eta }\right]\right\}$$

**FIGURE 8.**
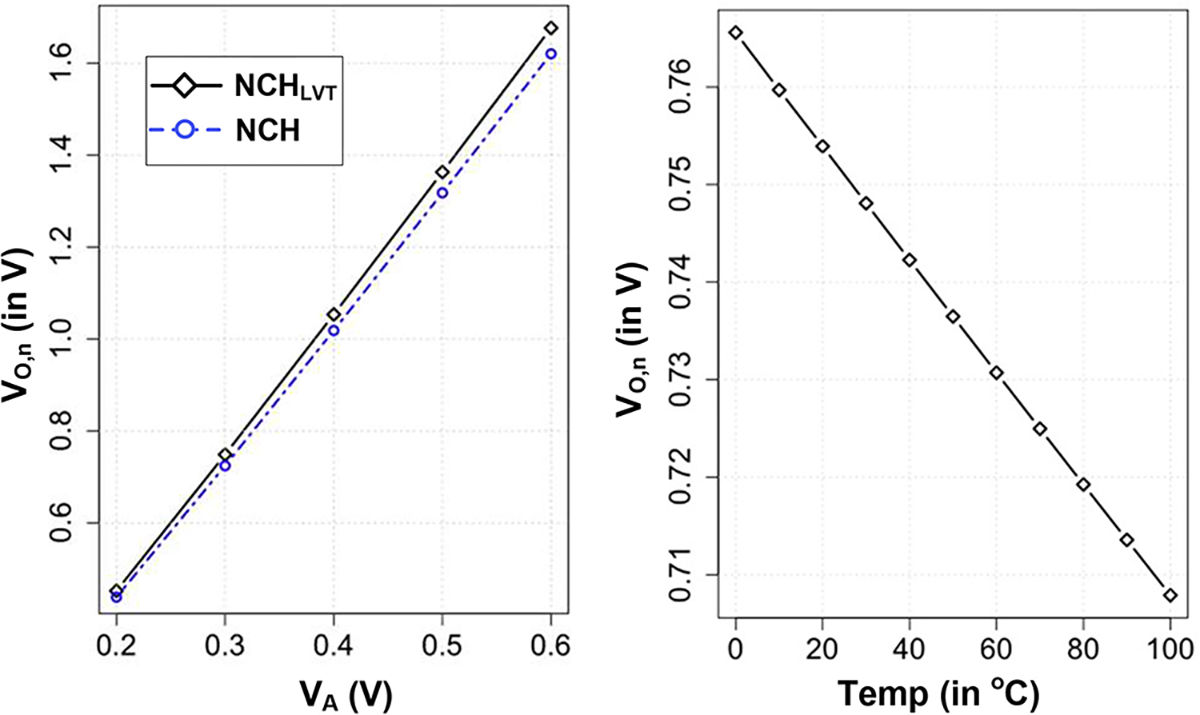
No-load output voltage of a multi-stage rectifier with input voltage and temperature variation.

Fig. 8a shows the simulation result of a 2-stage rectifier providing no-load output voltage for NCH and NCH_LVT_ transistor type. It shows the expected linear behavior with *V*_*A*_ with only 3% difference in output voltage. Note that the body effect of transistors in a multi-stage design also does not directly affect the open circuit voltage. Fig. 8b shows the temperature variation of *V*_*O*,*n*_ which shows the anticipated CTAT behavior similar to a single stage design.

#### Loaded Output Condition:

To analyze the loaded output condition in a multi-stage rectifier, we rely on our half-wave rectifier analysis. Fig. 7 shows this circuit where the internal nodes are named to help understand the analysis. The nodes whose output is connected using a capacitor to an RF source are called *X*_*i*_ and the output node whose capacitors are connected to ground are called *O*_*i*_. The average voltage of each internal node are designated as *V*_*X*,*i*_ and *V*_*O*,*i*_ which are constituted out of half-wave stages. Node *X*_*n*_ connects the final half-wave stage to the output whose average voltage is given by *V*_*X*,*n*_. The output voltage is given by *V*_*O*,*n*_ and it sees the output load current *I*_*O*_. When a positive voltage is applied at *X*_*n*_, it will see *k* · *V*_*A*_ + *V*_*X*,*n*_ which will be greater than *V*_*O*,*n*_ and supplies current to the output. When the negative voltage is applied at *X*_*n*_, it will see *V*_*X*,*n*_ − *k*·*V*_*A*_ and the transistor *M*_*n*_ gets reversed biased supplying very small leakage current to the output in this stage. However, *M*_*Xn*_ gets forward biased in this phase, which supplies the charge in the negative phase to maintain the average voltage *V*_*X*,*n*_. Owing to this scenario, average voltage across each half-wave stage, *V*_*D*_ would be approximately be equal. In reality, there will be some difference between the voltages each half-wave stage but it is ignored for this analysis. For an *n*-stage rectifier, there are 2*n* half-wave stages, therefore, *V*_*O*,*n*_ = 2*n*·*V*_*D*_. This sets the value of *V*_*X*,*n*_ = *V*_*O*,*n*_−*V*_*O*,*n*_/2*n*.

Therefore, when *M*_*n*_ is forward biased, it sees gate-source voltage of *V*_*X*,*n*_+*k*·*V*_*A*_−*V*_*O*,*n*_ = *k*·*V*_*A*_−*V*_*O*,*n*_/2*n*. The average current coming out of *M*_*n*_ is the output load given by

(17) $$I_{O}=\frac{I_{ST}}{2}\left[e^{\left(k\cdot V_{A}-V_{O,n}/2n\right)/\eta V_{t}}-1\right]$$

The output power in this case is given by

(18) $$P_{O}=V_{O,n}\cdot \frac{I_{ST}}{2}\left[e^{\left(k\cdot V_{A}-V_{O,n}/2n\right)/\eta V_{t}}-1\right]$$

Maximizing this expression with respect to *V*_*O*,*n*_ gives the expression for voltage corresponding to maximum power,

(19) $$V_{max,n}=2n\cdot \eta \cdot V_{t}\left[1-e^{-\left(k\cdot V_{A}-V_{max,n}/2n\right)/\eta V_{t}}\right]$$

**FIGURE 9.**
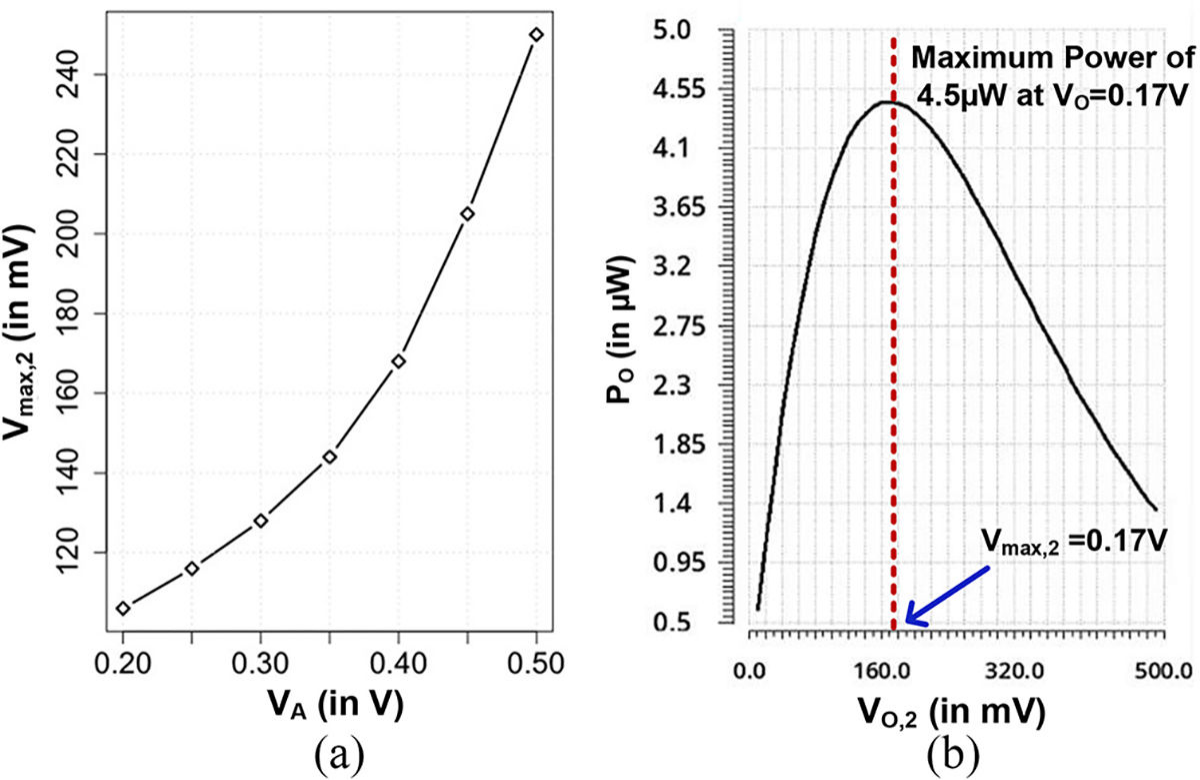
Simulation result showing variation of maximum power point in a 2-stage rectifier. a) Variation of maximum power point with *V*_*A*_, b) simulation showing output power as a function of output voltage *V_O,n_*.

Eq. (19) is similar to eq. (10) and similar to half-wave rectifier, multi-stage rectifiers will also have linear increase with *V*_*A*_ in the saturation region, while eq. (19) relates the behavior in the sub-threshold region. Fig. 9a shows the variation *V*_*max*,*n*_ with *V*_*A*_ in a 2-stage rectifier showing a similar trend. Fig. 9b shows the variation of output power as a function of output voltage clearly showing a peak response. We also note that the output power increases as with transistor’s drive strength. Since the output power directly depends on transistor’s drive strength, body-effect in a multi-stage design will adversely affect rectifier’s efficiency. Also deep *n*-Well transistors are commonly used to alleviate the body effect.

### C. STAGE-SELECTION IN MULTI-STAGE RECTIFIER

Stage-selection requirement for the no-load condition would require a design that can realize the maximum output voltage but the strategy diverges for the loaded condition. For a given transistor type, increasing the drive current increases maximum output power in half-wave and multi-stage rectifier. In this section we analyze, how the maximum power varies, both with transistor size and number of stages. In our previous analysis, the input resistance of the source is not included as the analysis directly focused at the input voltage level *V*_*A*_. However, as we move our analysis towards maximum obtainable power, source resistance has to be factored. In this case, 50Ω series resistance is used to model an antenna. Further, we have assumed that an input voltage 400mV with a source resistance of 50Ω drives the rectifier. The bondwire, pad, and ESD parasitic were included into a 2nH inductance and 200fF capacitance at the input.

**FIGURE 11.**
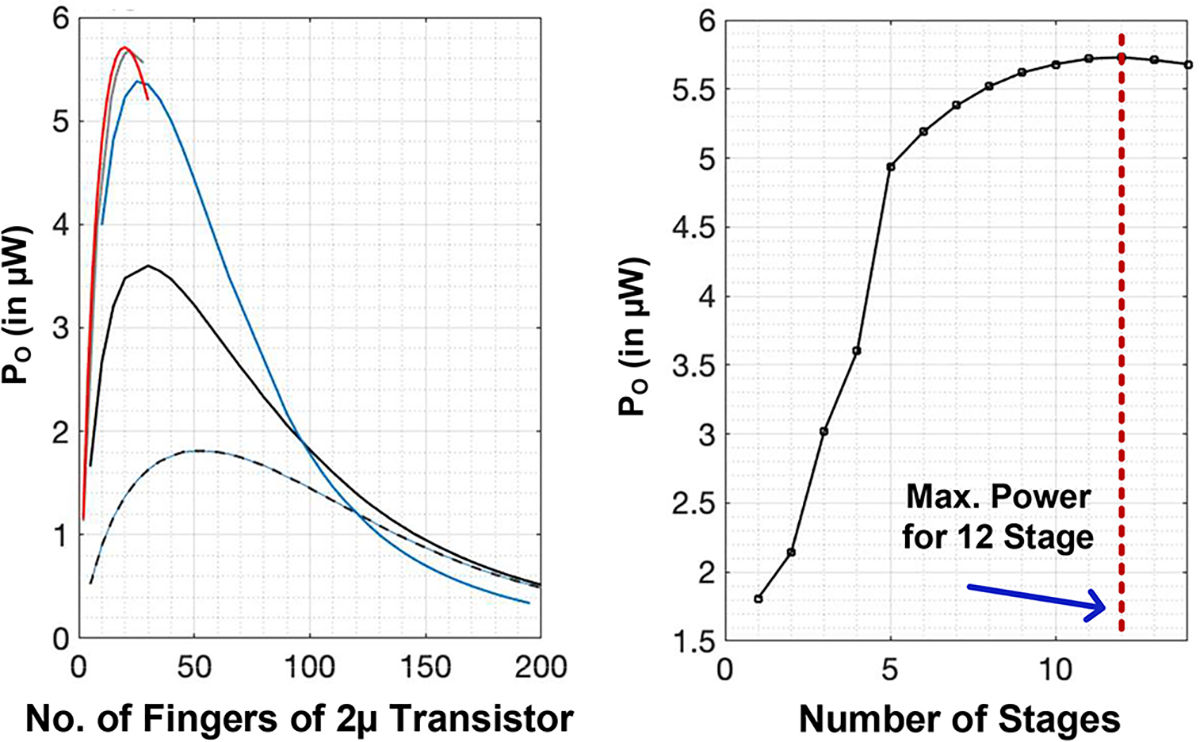
Simulation result to obtain maximum power by sweeping device size and number of stage for a multi-stage rectifier design for a 400*mV* input voltage.

We earlier showed that a fixed output voltage, *V*_*max*,*n*_ is obtained for the maximum power. Consequently, transistor size can be increased to obtain more power by increasing the current. However, this will reduce the level of *V*_*A*_ seen at the input. For an *n*-stage rectifier, an optimal device size exists for maximum output power. However, as we change the number of stages, the output power and device size will also change. This requires a nested sweeping of transistor size and number of stages to obtain the maximum power for a given input power. Fig. 11a shows maximum output power variation with device size. We swept the device width from 2 – 400*μm* for different *n*-stage rectifier. As the number of stages change, the maximum output power changes and a peak power is realized for different device size inline with eq. (19). We simulated the design for a 400*mV* input voltage. A 12-stage rectifier with a width of 40*μm* yielded the maximum power. As the number of stages increased, maximum power increased up to 12-stages after which it started decreasing (Fig. 11b). The maximum achievable efficiency for this circuit is 11.56%.

#### Role of Matching Network:

Eq. (19) clearly indicates that the maximum power increases with an increase in the input voltage swing. The circuit configuration with antenna and rectifier will keep the voltage swing below the received voltage swing due to the resistive drop. However, we can bring a reactive component to the mostly capacitive rectifier by introducing the matching network. Assuming that each rectifier diode stage presents a parasitic capacitor *C*_*PD*_, the input of the rectifier presents a total capacitance of *n*·*C*_*PD*_||*C*_*n*_. As the capacitor *C*_*n*_ ≫ *C*_*PD*_, the total capacitance presented by the rectifier is approximately equal to *n*·*C*_*PD*_. This will get added to the parasitic capacitor of bond-pads, ESD circuits, and board parastics to yield an equivalent capacitance of *C*_*EQ*_. A single inductor matching will yield the input voltage *V*_*I*_/*R*·1/*jωC*_*EQ*_. It clearly shows that reducing the *C*_*EQ*_, gives higher voltage at the input of the rectifier. A smaller transistor, a fewer number of stages, and lower parastics will give higher voltage and hence higher efficiency for the design. However, the high quality factor inductor would reduce the total bandwidth of the matching network. In later sections, we will outline the design principles for matching network, role of parasitics, and the optimal device size.

The issue comes up when most energy harvesters adopt *n*-MOS transistors rather than *p*-MOS due to its better current driving ability with the same size ratio, but the body of NMOS have to be tied to ground hence *V*_*SB*_ increases as source increasing the body effect.

## III. COMMON RECTIFIER TOPOLOGIES

Apart from the conventional half-wave topologies discussed in the previous section, bridge-type full-wave rectifiers are also commonly used for energy harvesting applications.

### A. BRIDGE-TYPE FULL WAVE RECTIFIER

**FIGURE 12.**
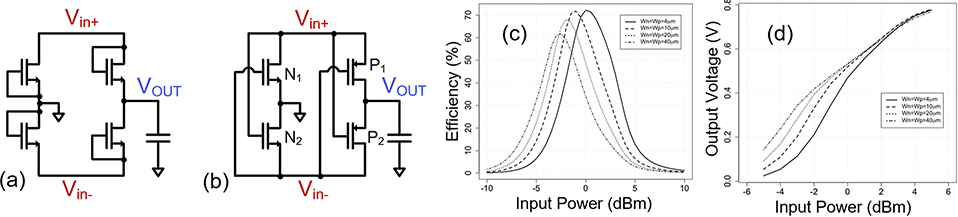
Schematic of a (a) bridge-type full wave rectifier, a (b) cross connected bridge rectifier, (c) variation of efficiency with received input power and transistor size for a single stage cross-connected bridge type rectifier (*L*_*n*_ = *L*_*p*_ = 60*nm*, *R*_*L*_ = 25*kΩ*), and (d)variation of output voltage *V*_*OUT*_ with received input power and transistor size for a single stage cross-connected bridge type rectifier (*L*_*n*_ = *L*_*p*_ = 60*nm*, *R*_*L*_ = 25*kΩ*).

Full-wave bridge rectifier which is comprised of four diodes and one capacitor is a popular topology. A MOS rectifier structure can thus be formed using only *n*-MOS and/or *p*-MOS devices. *V*_*TH*_ of MOS transistors will reduce the efficiency of the rectifier. Fig. 12a shows the circuit design of a single stage bridge type rectifier. It conducts current in both phases. The analysis presented in Section II applies for this circuit as well while taking into account conduction in both phases. Note that the no-load output voltage for this circuit will have similar behavior as the half-wave rectifier (eq. (7)). This is because when one set of diodes conduct in the positive phase, the other set will have leakage and a similar operating condition is realized. In case of loaded output condition, the primary design goal is to maximize efficiency. Note that in a full-wave rectifier, the output current increases but so does the associated switching and conduction losses. The efficiency optimization therefore requires proper device sizing for a given operating condition. Apart from device sizing, another general idea to improve efficiency includes improving diode characteristics through circuit design techniques.

### B. CROSS-CONNECTED BRIDGE RECTIFIER

A popular method for improving the performance of a bridge-type rectifier is to reduce its forward voltage drop by using a cross-connected bridge rectifier as shown in Fig. 12b. This type of rectifier uses internal nodes within the rectifier to bias the transistor gates. During positive half-cycles of the input voltage, current flows through P_1_ and N_2_, while transistors N_1_ and P_2_ are in the cutoff region. The situation is reversed for negative half-cycles of the input voltage. In each case, the cross-coupled PMOS transistors (i.e., P_1_ or P_2_) have higher *V*_*GS*_ (compared to a diode-based rectifier), therefore lower on-resistance. Due to this self-driven scheme, the cross-connected can operate at lower RF input power levels. To maximize efficiency, the body terminals of the *p*-MOS and *n*-MOS transistors should be connected to *V*_*OUT*_ and ground, respectively. One major drawback of this design is that for input voltages larger than *V*_*TH*_, the transistors do not turn off immediately after *V*_*in*_+ drops below *V*_*OUT*_. Yet, it is one of the commonly used types of topology. The capacitors need to be large enough such that their impedance is small compared to the on-resistance of the MOS transistors. Therefore large transistors lead to large coupling capacitors, which can result in higher area overhead.

Additional design consideration comes from the parasitic device capacitance between input and output. It provides an AC bypass of the input power to the output. In other words, it divides part of the input power and send it directly to the output without rectifying. That results in a reactive power component at the output which will reduce the efficiency of the rectifier. Essentially AC bypass of input through the parasitic series capacitor and rectification by the diode depends on the size and I-drive of the rectifier. If we keep the size of the transistor small, AC bypass will be small, however the rectification will also be small. Therefore, transistor size should increase to increase the rectification of input power. However, after a point increasing the size only increases the output power incrementally but it increases the bypass AC component linearly. Therefore, the AC-bypass due to parasitic capacitor also requires optimal device sizing. It will also impact the matching network design for the rectifier as it creates an output power component which is reactive. Due to these factors, devices with higher drive current with low capacitance are preferred.

Fig. 12c shows efficiency variation with the transistor size and input power. It achieves peak efficiency at a given input power level for a given transistor size. By increasing the device size, peak efficiency can be realized at lower power levels. However due to the increase of overhead associated with the switching loss, the value of the peak decreases. The output load *R*_*L*_ connected to *V*_*OUT*_ in this case was 25*k*Ω. This behavior is also verified by later silicon implementation in Section V. Fig. 12d shows the variation of output voltage, *V*_*OUT*_ with transistor sizing and input power at same output load. *V*_*OUT*_ value is higher as input power increases. However, it starts saturating at higher input power level due to conduction in the reverse-biased condition.

## IV. OTHER DESIGN CONSIDERATIONS

Recent works focus on designing the efficient RF energy harvesting system under low input power conditions. The biggest challenge is to realize high PCE in small form-factor at low input power level. The practical trade-off often limits the improvements in all aspects. In this section, different design scenarios are discussed in order to fairly evaluate them. Fig. 1 shows a typical structure of a energy harvesting system. Designs in [21]–[23] also use a chain of rectifiers to start up the entire system from initial state and maximum-power-point tracking (MPPT) is implemented to extract the maximum power. Since DC-DC converter can achieve 80 – 90% efficiency to step up/down voltage, the rectifier does not need extra stages at the cost of decreasing PCE. In the IMD application, the major design difference from above is to realize high integration with small form-factor. The challenge is to design an energy harvester which could provide high current injection, high PCE with limited silicon area.

### A. GENERAL EFFICIENCY OPTIMIZATION DESIGN FLOW

Important goals when designing an energy harvesting circuit is to realize high PCE or voltage conversation ratio (VCR). The VCR is defined as the ratio of the rectified output DC voltage (of the last stage) to the peak amplitude of the input AC voltage of the rectifier (of the first stage). Unfortunately, techniques used to increase PCE often decrease the VCR. We can prioritize only one for a particular application. We detail an iterative design optimization flow for a rectifier design.

1. The first step is to assign or estimate the design constraints such as the resonant frequency, the output load (an RC model can be used to represent it), and the input voltage swing. The needed output capacitance should also be known early-on during the design optimization limited IC area for an implant.
2. The suitable rectifier topology should be chosen based on application needs, as the rectifier type that displays the highest PCE depends on various parameters such as frequency, input power, transistor size, and desired VCR. Here our analysis serves as the basis for choosing initial device size, type, and number of stages.
3. The third step involves maximizing the PCE. In most rectifiers, this is achieved by finding the optimal transistor width. After the first cut size is provided by our analysis, it is important to simulate the design with the target load for final optimization. As our analysis shows that there is a load value that gives a peak efficiency for a given size (Fig. 9), conversely a device size exists that gives peak efficiency for a given load.
4. Step 3 is repeated in order to find the optimal capacitance and rectifier width simultaneously. Afterwards, the layout is drawn to extract the parasitics which are needed to obtain a more accurate design.
5. Finally, the input impedance of the rectifier is simulated to design the matching network. Note that the matching network has to be tuned such that *V*_*RF*_ is achieved. This often means that maximum efficiency is obtained by finding the sweet spot between impedance matching and desired voltage swing.

### B. PERFORMANCE COMPARISON

**FIGURE 13.**
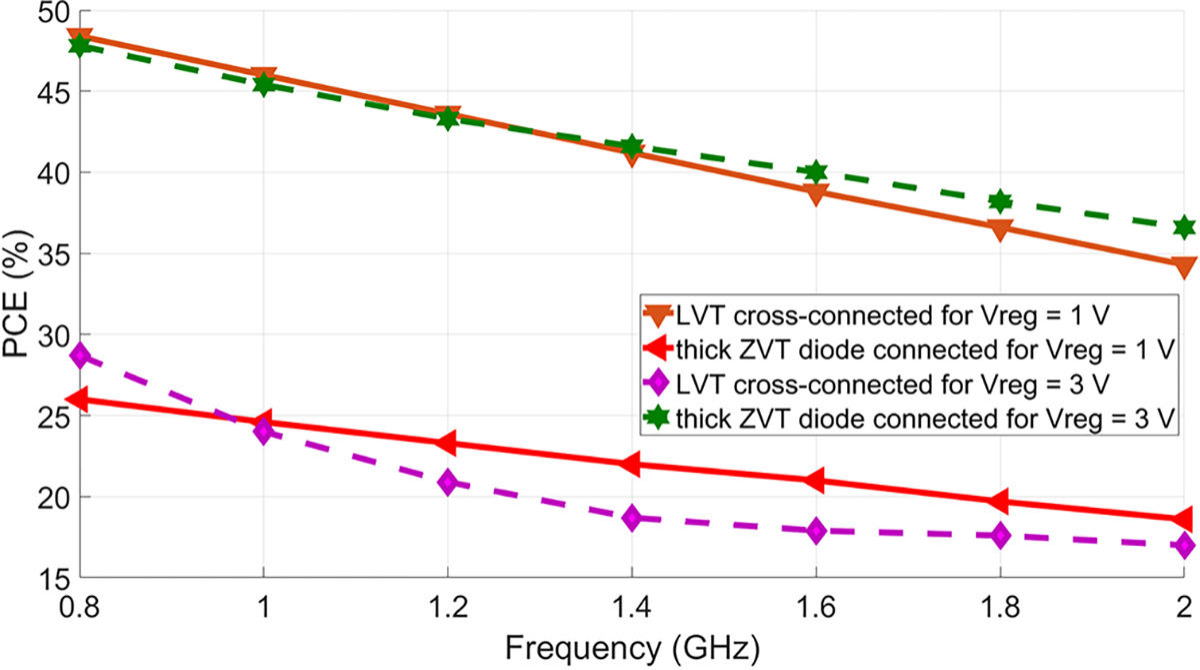
Post-simulated PCE over frequency of the 2 rectifier topologies with a load of 30kΩ and a rectified voltage of 1 V and 3 V.

To demonstrate how changing the target rectified voltage (and thus power delivered to the rectifier) changes the optimal topology, we compare *L*_*VT*_ cross-connected topology with a *Z*_*VT*_ diode-connected topology. For each topology, the transistors were sized for maximum PCE. Fig. 13 shows that for a rectified voltage of 1V, the cross-connected bridge rectifier with *L*_*VT*_ transistors shows a higher PCE compared to the diode connected topology *Z*_*VT*_ transistors. The opposite is true for a regulated voltage of 3 V. It can be concluded that cross-connected topology is more efficient while producing low output voltage.

### C. MATCHING NETWORKS

#### 1) SIGNIFICANCE

The matching network is used for directing maximum AC power towards the rectifier at one or more RF frequencies. In addition, they can also boost the voltage through a passive gain and can act as a low pass filter to reject higher order harmonics generated by the rectifier which can get reradiated. Often, published results on rectifier efficiency are calculated mathematically from the measured S-parameters in a way that does not include input mismatch power losses. Although PCE is important to consider, it does not give a good idea of how the rectifier performs in a practical circuit. Co-designing the matching network and the PCE of the rectifier is crucial for maximizing the power harvesting efficiency of the system. Some rectifier architectures provide a very large PCE at the expense of a very large input impedance which makes it difficult to design a small area matching network.

#### 2) FIXED MATCHING SYSTEM

The matching network is one of the last blocks to design in the energy harvesting systems because it depends on the input resistance of the rectifier (*R*_*IN*_) which is linked to the power as,

(20) $$R_{IN}=V_{A}^{2}/\left(2P_{IN}\right)$$

where *V*_*A*_ is the input amplitude and *P*_*IN*_ is the input power.

To maintain a low loss for matching, reactive matching networks are used. Common matching networks are typically composed *LC*-networks such as *π* and *T* networks. High quality factor (*Q*) matching networks provide a passive voltage gain to increase the voltage swing at the input are desirable to minimize loss, however, they suffer from narrow bandwidth making it sensitive to frequency variations. This is important when using on-chip passive elements whose values can change due to process.

An off-chip matching network provides additional design flexibility while supporting higher *Q* and larger component values. Unfortunately, off-chip components prevent the miniaturization of the implant and therefore are not always feasible. On the other hand, it is important to note that IMDs limited to *mm*-dimensions will not always have the space to include on-chip matching networks as large inductors are needed.

#### 3) ADAPTIVE MATCHING SYSTEM

The rectifier operates in different regions since the input sees a large AC signal without any DC bias. This results in an impedance variation making it difficult to match at all times. Impedance variations also happens due to changes in received power and output load. Often matching for worst-case scenarios, i.e., when *V*_*A*_ is lowest. If variations are large, then a wideband impedance matching network is used at the expense of a low *Q*-factor.

An alternative method is to include an auto-tuning impedance matching network that maintains a constant efficiency even when the received RF power or the load changes [24]–[26]. In [27], authors presented an adaptive antenna-impedance tuning unit for ULP IMDs operating at 2.4GHz. The design requires a power amplifier, a tunable matching network, a capacitive attenuator, a mixer, and a *g*_*m*_-C filter. In [24], authors developed a fully integrated wireless power transfer receiver with a synchronous adaptive matching network that guarantees the maximum achievable efficiency for a given load. A switched capacitor array was controlled according to a sign-based gradient descent algorithm to maximize the power delivered to the rectifier load at the receiver. These techniques address the challenge by adding a processing unit for dynamic calibration. However, the additional circuitry needed to measure the impedance of the rectifier or that of the network increases the power consumption and area and can be used only if a significant efficiency increase can be realized.

### D. MEASUREMENT TEST-BENCH SETUPS

#### 1) IMPACT OF BONDPADS ON THE PCE

A bondpad is equivalent to a shunt capacitor which becomes a low-impedance path at high frequency. Table 2 presented the parasitics of different sizes of IO pads. The conventional size bond pads connected to the input of a rectifier could severely reduce PCE. This effect may be minimized by decreasing the size of the bond pads and by using octagonal shapes. Furthermore, relying on precision RF probes instead of wirebonding allows further reduction of the input bondpad area.

Bondpads typically include diodes in order to shield against ESD damage. However, the diodes can leak a significant amount of RF current. Fortunately, due to the nature of rectifiers, the ESD structure can be smaller or not needed as in rectifiers the gate terminals are also connected to source/drain regions of other transistors providing a path for ESD charge.

#### 2) IMPACT OF EMI ON THE MEASUREMENT SETUP

**FIGURE 15.**
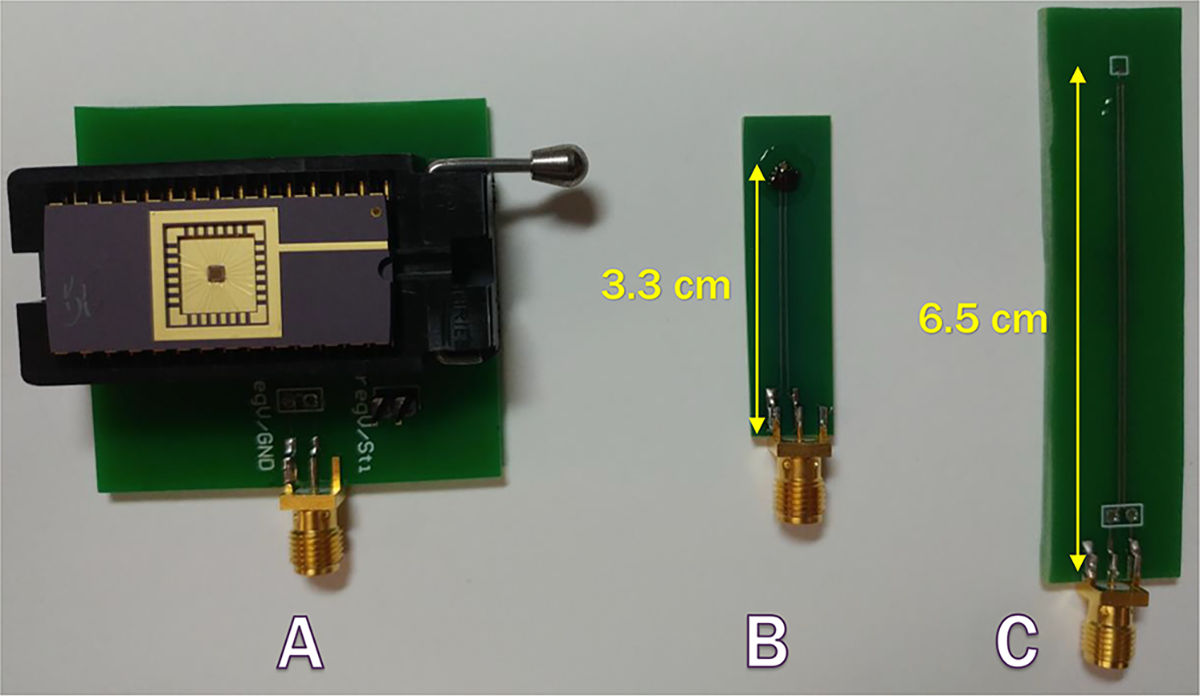
Picture of different PCB setups used to measure the regulated voltage generated by the Rx device [28].

When transmitting large amounts of power at a short distance, the setup becomes affected by unwanted eddy currents in the PCB traces [29]. Eddy currents loss can be minimized in the silicon substrate by using a silicon-on-insulator (SOI) process [30], however, eddy currents in highly conductive PCB traces are difficult to minimize. A common solution to this problem is to cover the PCB with a high-permeability ferrite sheet which deflects the magnetic flux. Unfortunately, commercial ferrite shields are not effective at high frequencies in which *μ*m-sized coils need to operate in order to resonate. One method is the careful design of the PCB layout. The design techniques that have been applied include: i) reducing the area of closed metal loops as much as possible, ii) keeping the number of traces on board to a minimum, iii) reducing the width of the metal traces to a minimum, iv) using coaxial cables to interface with the PCB, v) prioritizing SMA connectors over headers or large BNC connectors and placing them far from the transmitter coil, and vi) prioritizing wire-bonded dies. Fig. 15 shows the our test boards designed specifically to minimize the EMI. The induced AC voltages at the output of 3 identical rectifiers from a wireless transmitter have been measured and is found to be 900, 200 and 50 mV, for setups A, B, and C, respectively. EMI in setup B is larger than that of setup C because of the longer PCB traces. Setup A has the largest recorded EMI due to the use of DIP packaging. The Tx coil was placed 5 mm away and was transmitting a 35 dBm 1 GHz power signal. Increasing transmitted power and reducing the Tx-Rx distance increase the effect of EMI.

## V. MEASUREMENT RESULT

To verify our analysis and design principles discussed in Section II, we carried out three different chip designs. We designed 10-stage Dickson rectifier circuits in 65nm and 130nm CMOS technologies and a 3-stage cross connected bridge rectifier in 180-nm SoI technology. Measurement setup is presented in Fig. 17. For the open circuit output voltage measurement, a buffer is used to prevent from loading effect of the oscilloscope and the output voltage is read from oscilloscope for loaded Dickson rectifier and cross-connected rectifier while the input power of the rectifier is the product of the power given by the signal generator and the reflection coefficient measured by Vector Network Analyzer (VNA). The purpose of 10-stage Dickson rectifier was to verify no-load/light load output condition while cross coupled design was carried out for maximum efficiency design.

### A. SINGLE-ENDED CONVENTIONAL DICKSON RECTIFIER

**FIGURE 16.**
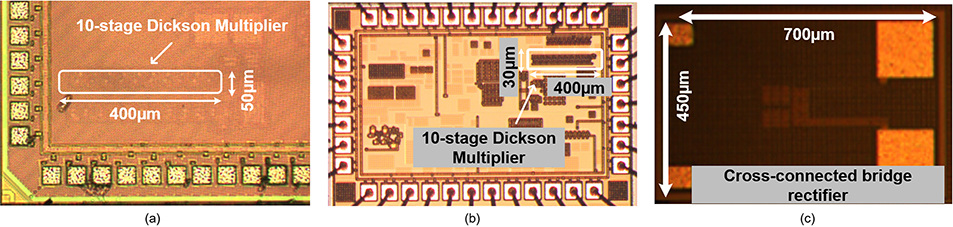
(a) Die micro-graphs of a 10-stage Dickson-multiplier rectifier in 65-nm, (b) 130-nm CMOS technology and (c) a cross connected bridge rectifier in 180-nm technology.

**FIGURE 17.**
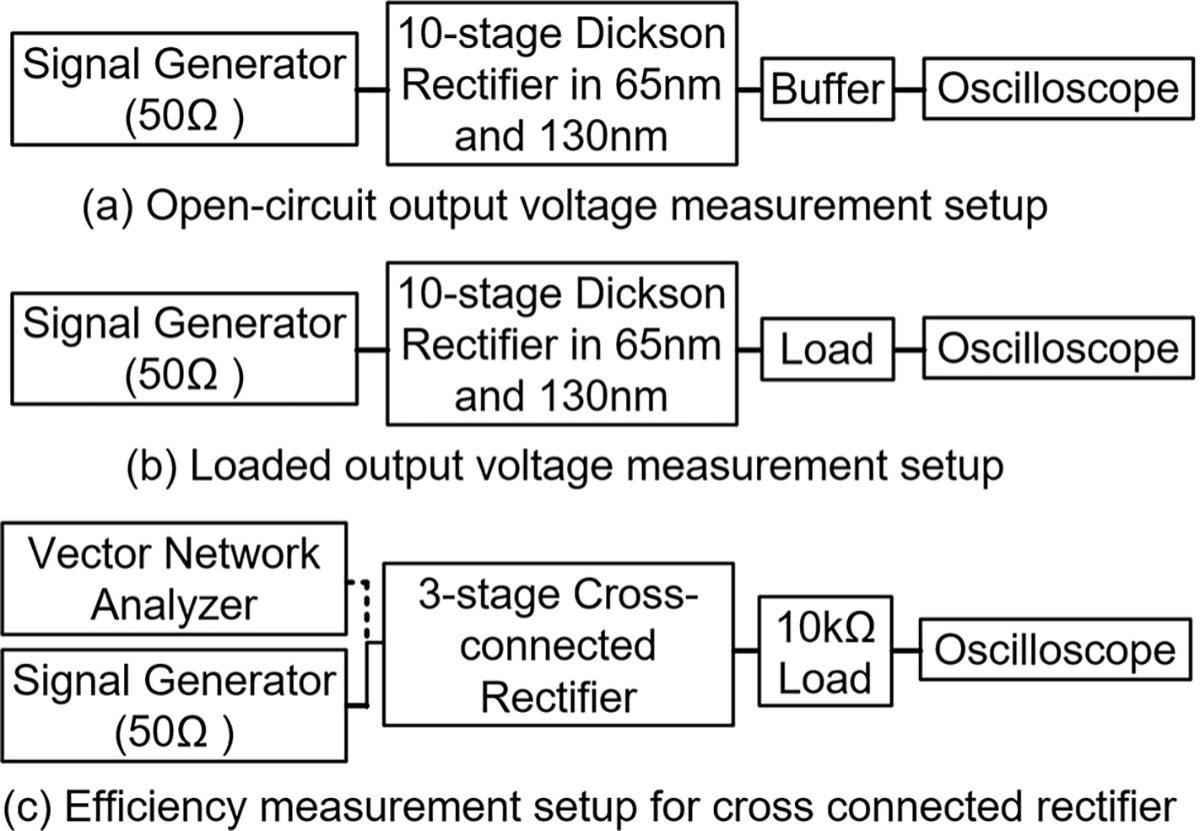
Bench-top measurement setup to measure the open circuit voltage and efficiency of rectifier circuits.

The 10-stage rectifier circuits were developed with minimum sized transistors in each of 65nm and 130nm technologies. For the 65nm rectifier design, we used *n*-MOS transistor size of 200nm/60nm while the 130nm design used *n*-MOS transistor size of 160nm/120nm. Both designs use approximately 200*f*F metal-insulator-metal (MIM) capacitors to implement the rectifier capacitors. Fig. 16 shows the die-micrograph of the two designs. Fig. 16a shows the die-micrograph of the 65nm design which has an area of 400 × 50*μm*^2^. Fig. 16b shows the die-micrograph of 130nm design and it has an area of 400×30*μm*^2^. 130nm technology offers higher density MIM capacitor and consequently has lower area. Fig. 16c shows the die-micrograph of 3-stage cross coupled design in SoI technology. This design included large bond-pads to be able to probe the rectifier inputs. Its size is 450 × 700*μm*^2^.

**FIGURE 18.**
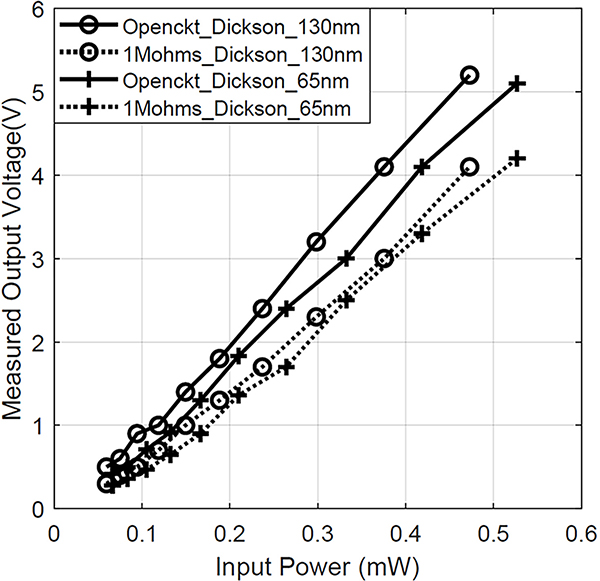
Measured output voltage comparison of traditional 10-stage Dickson multiplier at 950MHz between 65nm technology and 130nm technology.

The Dickson multiplier designs were additionally targeted to generate higher output voltage which is desirable for animal tissue stimulation. Our designs target a high output voltage from −10 dBm to 0 dBm incident power which is below the specific absorption rate (SAR) requirement mandated by FCC. Therefore we adopted 10 stages to amplify the limited input swing. Fig. 18 presents the measured output voltage at open circuit condition and at 1*M*Ω load condition of the Dickson multiplier manufactured in 65-nm (*V*_*TH*_ = 0.48*V*) and 130-nm CMOS (*V*_*TH*_ = 0.35*V*) technology respectively. The measured open circuit output voltage for both designs are close to each other and supports the notion that the technology or transistor type does not have a large impact on the open circuit output voltage as laid out in our analysis and eq. (19) with respect to no-load output voltage in a Dickson stage. It also shows a linear output voltage with respect to the input at the ultra-low-power condition which further verifies our analysis in Section II. The linear output of both rectifiers as well as their similar output voltages despite the technology difference agrees with our no-load analysis.

**FIGURE 19.**
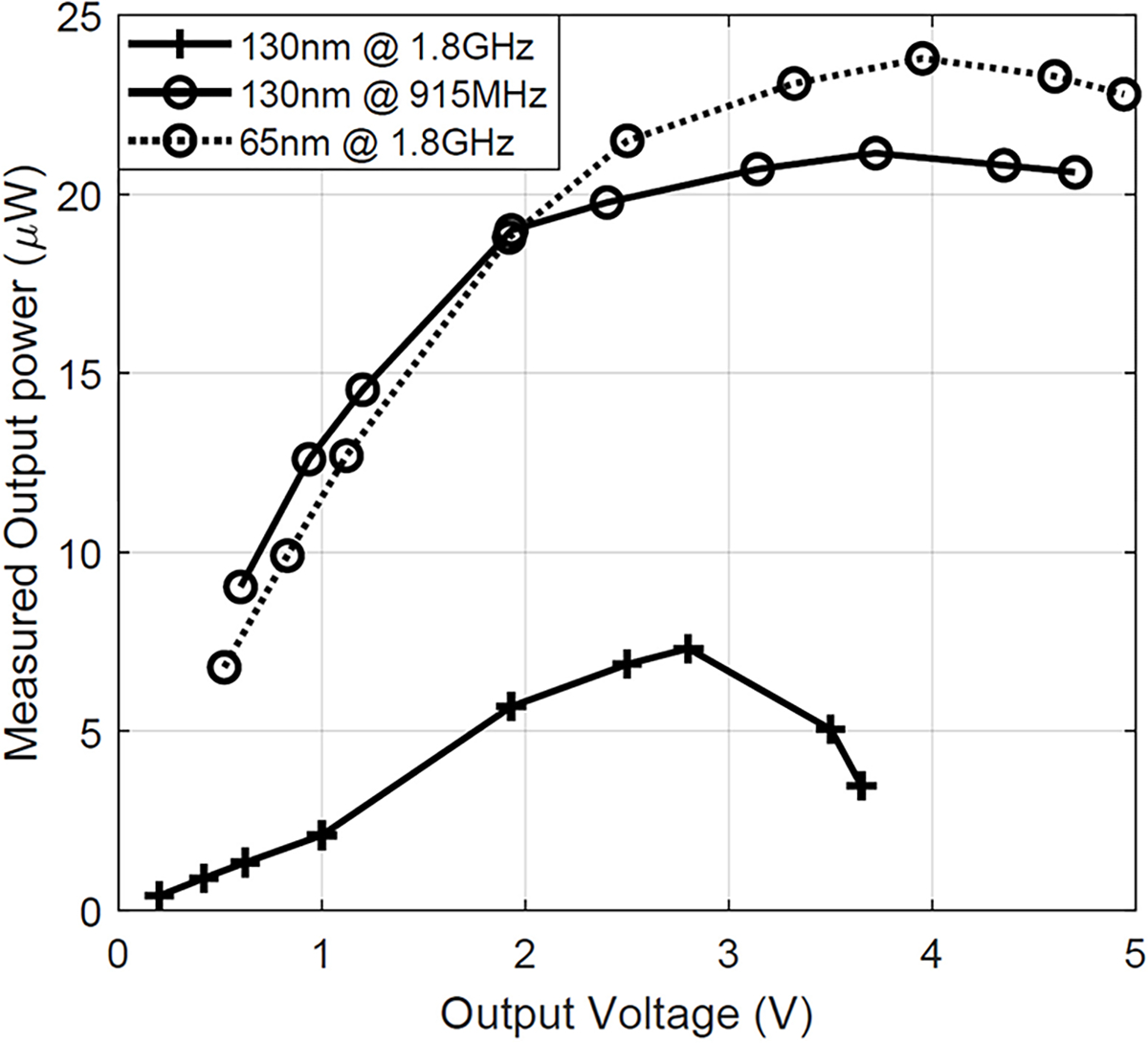
Measured maximum output power with different output voltage at −2 dBm input power.

As the 10-stage rectifier designs in both technologies were carried out with minimum sized transistors, they are not optimized for PCE. Fig. 19 demonstrates the received output power at fixed −2 dBm input power with different output voltage by changing the load resistance. It can be observed that there is an optimal output condition for each energy harvester design that draws the maximum power. Due to the existence of the ESD diode, the output voltage cannot go higher than 5V but this trend is still visible in the figure. After the peak-power, the output power decreases, a trend that is predicted by our maximum power analysis carried out in Section II. The measured output voltage trend agrees with eq. (9). This conclusion is meaningful because if the load condition of certain application is known beforehand, the energy harvester can be optimized to provide the most amount of power. It can also be noticed that, at the same received power level, the rectifier with 65nm outperforms the one with 130nm at high frequency, i.e., 1.8GHz due to the high driving current of 65nm transistor. Our analysis on loaded output condition also indicates that transistors with higher drive current will supply higher power. However, when the RF signal frequency goes down to sub-GHz, this advantage become less significant because while both devices are well matched, the input voltage amplitude is larger at low frequency than the voltage at high frequency, which contributes to the similar performance shown in the figure.

### B. CROSS CONNECTED BRIDGE RECTIFIER

**FIGURE 20.**
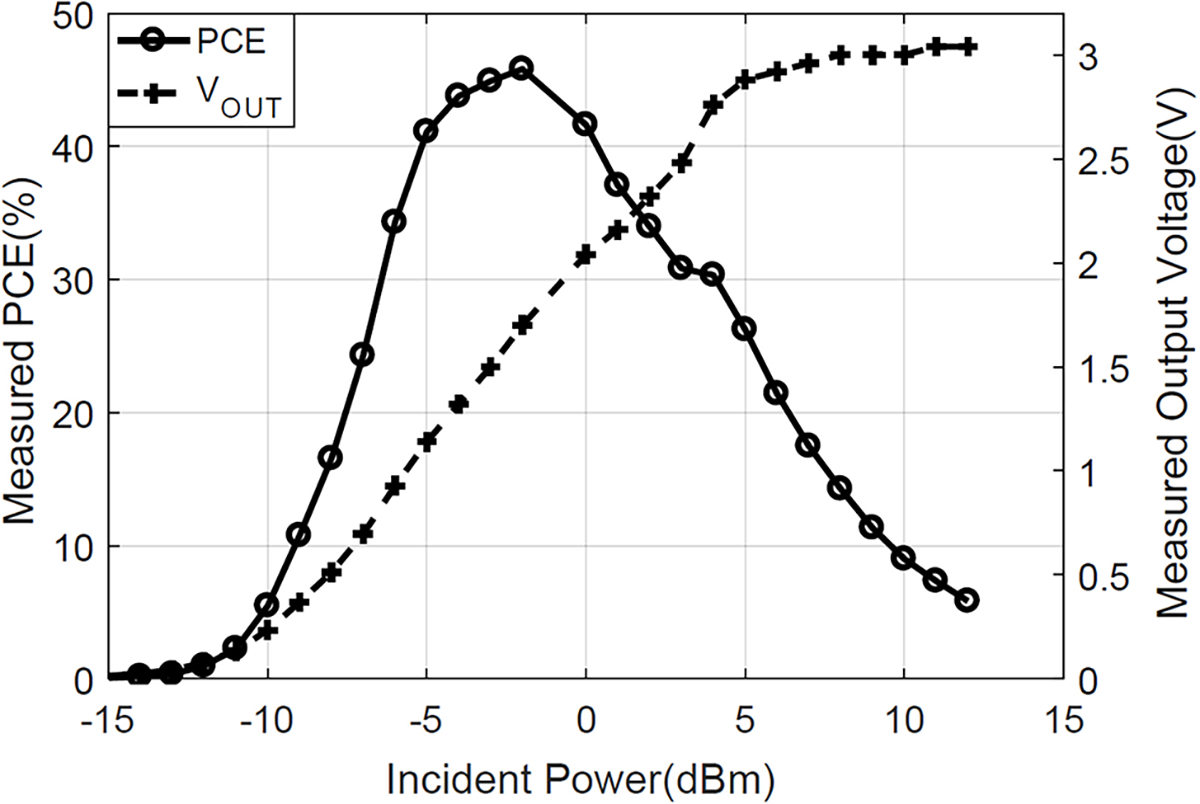
Measured rectifier efficiency and output voltage with 10 kΩ load resistor vs. incident power of the cross-coupled differential rectifier.

Following the design flow to optimize the parameters mentioned in the previous section, we implement the 3-stage conventional cross connected bridge rectifier in 180-nm SOI technology and its die photo is demonstrated in Fig. 16c. This design is optimized for maximum efficiency. The measurement results is shown in Fig. 20 which shows that we achieve a maximum PCE of 45% at −2 dBm. Further the peak power behavior also confirms our analysis carried out in Section II.

We further compared our 3-stage cross connected bridge rectifier in terms of PCE and other typical rectifier metrics with the state-of-the-art compact RF energy harvesting circuits in Table 3. For PCE, we mostly compare our 0.18*μ*m design with other designs. We achieve a peak efficiency of 45% which is among the best reported PCE. The peak efficiency is achieved at a 10*k*Ω load with −2dBm in power. This design is designed with a 50Ω termination so it doesn’t require a matching network. And our 10-stage Dickson rectifiers serve different purpose of high output voltage operation so it’s not included in the comparison table. Further, using eq. (15), peak PCE is given by 0.667·(1−*ηV*_*TH*_/*kV*_*A*_). Using the value of *V*_*TH*_ = 0.2 and *k* = 0.87 (obtained from simulations), the peak efficiency comes out to be 48%. Our peak efficiency results shows conformation with the mathematical derivation of efficiency in Section II.

## VI. CONCLUSION

This paper reviews and provides analysis for common circuit topologies for RF CMOS rectifiers for energy harvesting applications using square-wave approximation technique. Mathematical analysis of the operation of a half-wave and multi-stage *n*-MOS based rectifier, which is widely used in ultra-low-power energy harvesting applications such as IoT sensors, wearable applications, and miniaturized n-IMDs, is also presented in this paper. The analysis is conducted keeping in consideration that the RF power gathered in these applications is generally very low due to attenuation and propagation loss in the medium, and the relative small coil or antenna dimensions. Open circuit and loaded output conditions of the rectifier are discussed to meet different application requirements. The analysis shows that the output voltage in no-load condition does not depend on the transistor’s *V*_*TH*_. The square-wave approximation analysis also provided an upper limit of rectifier efficiency of 66.7%. The other design considerations like the stage selection, flow of designing the RF energy harvesting system, matching network design, the impact of bondpads ESD, and EMI effects are also covered in this paper. Measurement results are presented to show the characteristics of a 10-stage Dickson multiplier in 65nm and 130nm CMOS technologies and a 3-stage cross connected rectifier in 0.18*μ*m. Measurements of open circuit and loaded output condition were performed to verify analytical results. Measurement of open circuit voltage, output power characteristics, and peak efficiency show agreement with the theoretical analysis. Further these design methods were used to achieve a 45% efficient rectifier which is in close agreement of our mathematically obtained peak efficiency of 48%.

## Figures and Tables

**FIGURE 10.**
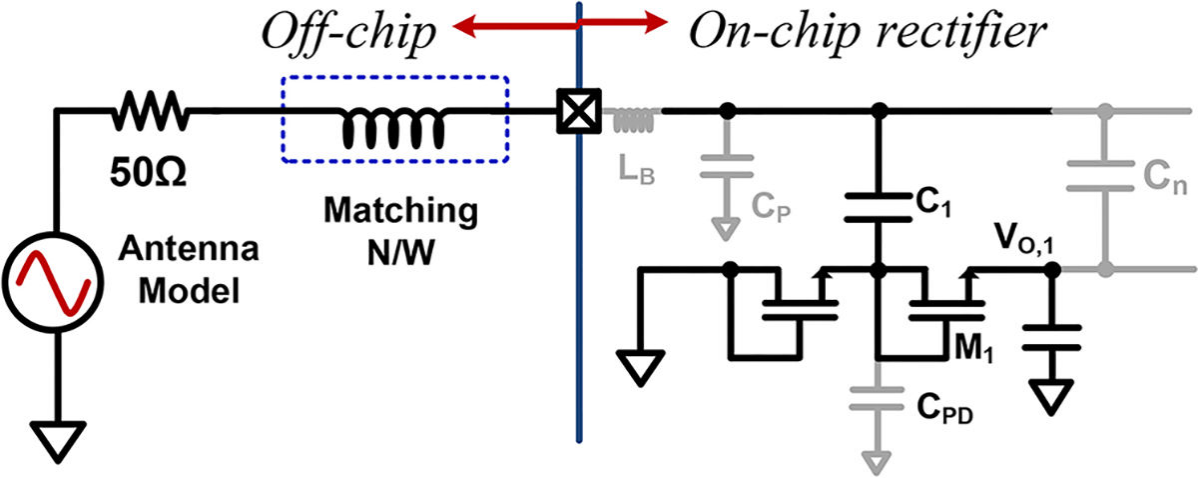
Block diagram of the multi-stage rectifier with matching network and interconnect parasitics.

**FIGURE 14.**
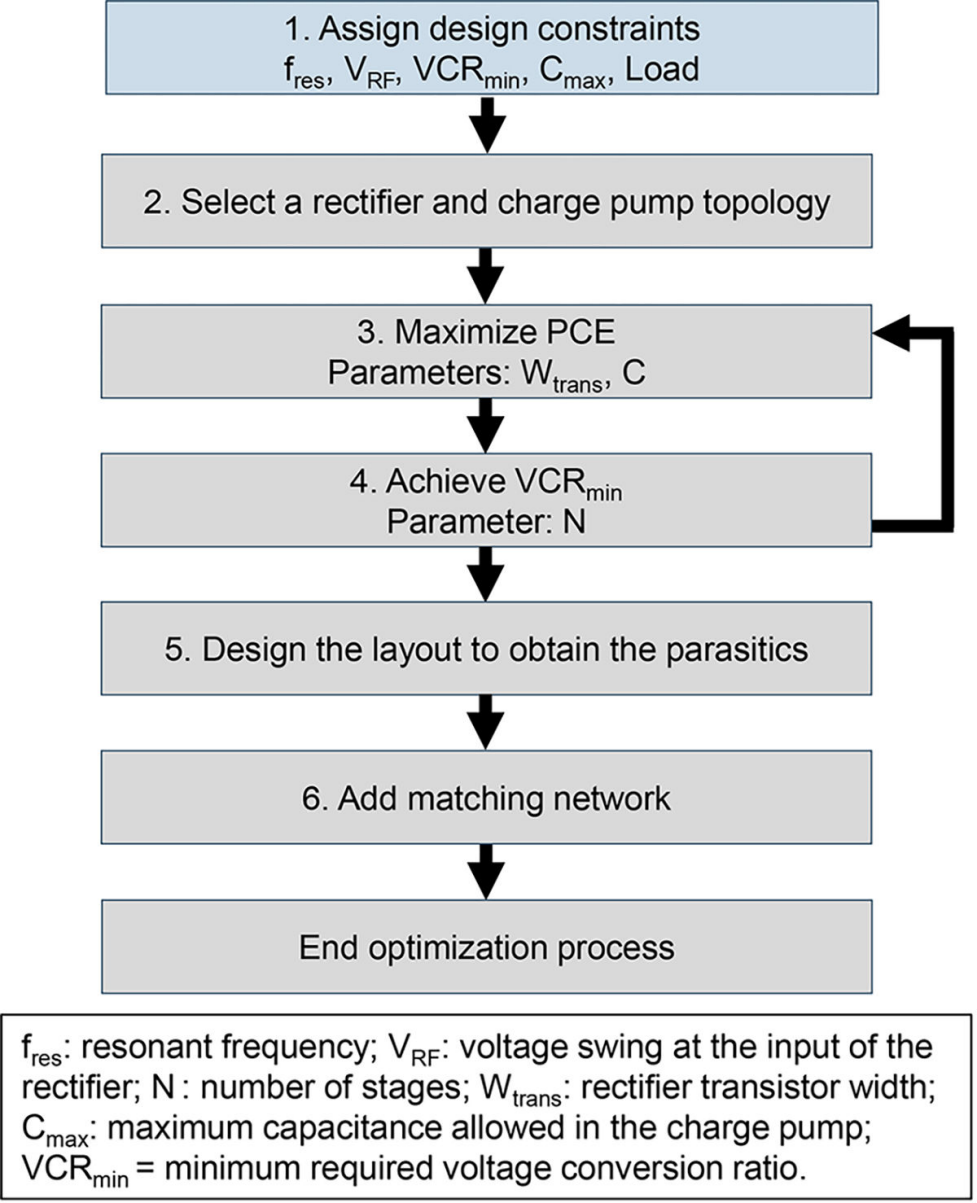
Charge pump rectifier iterative design optimization flowchart.

**TABLE 1.** Notations used in the paper.

Variable Name	Physical Definition
*V_t_*	Thermal voltage
*V_TH_*	Threshold voltage
*I_S_*	Sub-threshold saturation current
*η*	Ideality factor
*k*	Amplitude scaling factor
*V_ON_*	No-load output voltage
*T*	Time Period of RF power

**TABLE 2.** IO pads parasitics @ 915MHz from post-layout simulation.

Size (*μm*^2^)	Resistance (Ω)	Capacitance (fF)
50 × 50	0.91	117.5
80 × 80	0.90	278.5
100 × 100	0.51	415.6
120 × 120	0.58	580.0

**TABLE 3.** Performance comparison with previous energy harvesting circuits.

	TCAS-I’15 [13]	TCAS-I’17 [31]	JSSC’17 [32]	TBioCAS’18 [33]	JSSC’19 [34]	TCAS-I’19 [5]	JSSC’19 [7]	JSSC’21 [35]	**This work**
Technology	0.13*μ*m	0.13*μ*m	0.18*μ*m	0.13*μ*m	0.18*μ*m	0.13*μ*m	65nm	65nm	0.18*μ*m
Freq (GHz)	0.9 – 0.92	0.953	0.915	1.3	0.915	0.896	2.45	2.45	1.05
Area (mm^2^)	0.25	0.095	2.3×2.3	0.2×0.2	0.61×0.65	0.2×0.3	0.005×0.025	NA	0.315 (max.)
Application	IoT	IoT	IoT	IMD	IoT	IoT	IoT	IoT	Implants
Architecture	Adaptive threshold compensated	Differential cross-coupled	Reconfigurable 8-stage	Cross-connected bridge	Reconfigurable 12-stage	*V_TH_* compensated	Cross-coupled	Cross-coupled	Cross-connected
# of stages	12	3	1,2,4,8	4	2,3,4,6,12	4	1	1	3
Pin (*μ*W) @ Peak PCE	32	3162	1000	36	1260	316	500	38.9	631
Load @ Peak PCE	1*M*Ω	2*k*Ω	N/A	N/A	MPPT controller	300*k*Ω	PMU	PMU	10*k*Ω
Sensitivity	−20dBm @ 1V Vout	−8dBm @ 1V Vout wtih 50kΩ	−14.8dBm@ 1V Vout	N/A	−18.1dBm@ 1V Vout	−20.5dBm @ IV Vout	−22.7 dBm @0.4V Vout	−26.7 dBm @0.4V Vout	−10dBm @ 1V Vout with 470kΩ
MN	off-chip	none	on-chip	none	off-chip	off-chip	off-chip	off-chip	**none**
Peak PCE	32%	73.9%	25%*	38%	36%*	35.5%*	48.3%	32.3%	**45%**

* End-to-End efficiency including the reflection loss

## References

[1] Shafiee N, Tewari S, Calhoun B, and Shrivastava A, “Infrastructure circuits for lifetime improvement of ultra-low power IoT devices,” IEEE Trans. Circuits Syst. I, Reg. Papers, vol. 64, no. 9, pp. 2598–2610, Sep. 2017.

[2] Roy A , “A 6.45 μW self-powered SoC with integrated energy-harvesting power management and ULP asymmetric radios for portable biomedical systems,” IEEE Trans. Biomed. Circuits Syst, vol. 9, no. 6, pp. 862–874, Dec. 2015. 26731775 10.1109/TBCAS.2015.2498643

[3] Zhang Y , “A batteryless 19 μW MICS/ISM-band energy harvesting body sensor node SoC for ExG applications,” IEEE J. Solid-State Circuits, vol. 48, no. 1, pp. 199–213, Jan. 2013.

[4] Chen Y-P , “An injectable 64 nW ECG mixed-signal SoC in 65 nm for arrhythmia monitoring,” IEEE J. Solid-State Circuits, vol. 50, no. 1, pp. 375–390, Jan. 2015.

[5] Saffari P, Basaligheh A, and Moez K, “An RF-to-DC rectifier with high efficiency over wide input power range for RF energy harvesting applications,” IEEE Trans. Circuits Syst. I, Reg. Papers, vol. 66, no. 12, pp. 4862–4875, Dec. 2019.

[6] Karami MA and Moez K, “Systematic co-design of matching networks and rectifiers for CMOS radio frequency energy harvesters,” IEEE Trans. Circuits Syst. I, Reg. Papers, vol. 66, no. 8, pp. 3238–3251, Aug. 2019.

[7] Xu P, Flandre D, and Bol D, “Analysis, modeling, and design of a 2.45-GHz RF energy harvester for SWIPT IoT smart sensors,” IEEE J. Solid-State Circuits, vol. 54, no. 10, pp. 2717–2729, Oct. 2019.

[8] Khalifa A , “The microbead: A 0.009 mm^3^ implantable wireless neural stimulator,” IEEE Trans. Biomed. Circuits Syst, vol. 13, no. 5, pp. 971–985, Oct. 2019. 31484132 10.1109/TBCAS.2019.2939014

[9] Laiwalla F , “A distributed wireless network of implantable sub-mm cortical microstimulators for brain-computer Interfaces,” in Proc. 41st Annu. Int. Conf. IEEE Eng. Med. Biol. Soc. (EMBC), 2019, pp. 6876–6879. 10.1109/EMBC.2019.8857217 31947420

[10] Zhao Y, Rennaker RL, Hutchens C, and Ibrahim T, “Implanted miniaturized antenna for brain computer Interface applications: Analysis and design,” PLoS One, vol. 9, no. 7, 2014, Art. no. e103945. 25079941 10.1371/journal.pone.0103945 PMC4117534

[11] Yang K-W, Oh K, and Ha S, “Challenges in scaling down of free-floating implantable neural interfaces to millimeter scale,” IEEE Access, vol. 8, pp. 133295–133320, 2020.

[12] Moghaddam AK, Chuah JH, Ramiah H, Ahmadian J, Mak P-I, and Martins RP, “A 73.9% (LDCF) self-body-biasing technique for far-field RF energy-harvesting systems,” IEEE Trans. Circuits Syst. I, Reg. Papers, vol. 64, no. 4, pp. 992–1002, Apr. 2017.

[13] Hameed Z and Moez K, “A 3.2 V −15 dBm adaptive threshold-voltage compensated RF energy harvester in 130 nm CMOS,” IEEE Trans. Circuits Syst. I, Reg. Papers, vol. 62, no. 4, pp. 948–956, Apr. 2015.

[14] Lau WWY, Ho HW, and Siek L, “Deep neural network (DNN) optimized design of 2.45 GHz CMOS rectifier with 73.6% energy harvesting,” IEEE Trans. Circuits Syst. I, Reg. Papers, vol. 67, no. 12, pp. 4322–4333, Dec. 2020.

[15] Nakamoto H , “A passive UHF RF identification CMOS tag IC using ferroelectric RAM in 0.35-μm technology,” IEEE J. Solid-State Circuits, vol. 42, no. 1, pp. 101–110, Jan. 2007.

[16] Yi J, Ki W-H, and Tsui C-Y, “Analysis and design strategy of UHF micro-power CMOS rectifiers for micro-sensor and RFID applications,” IEEE Trans. Circuits Syst. I, Reg. Papers, vol. 54, no. 1, pp. 153–166, Jan. 2007.

[17] Gao H, Matters-Kammerer M, Milosevic D, Linnartz J-PMG, and Baltus P, “A design of 2.4GHz rectifier in 65nm CMOS with 31% efficiency,” in Proc. IEEE 20th Symp. Commun. Veh. Technol. Benelux (SCVT), 2013, pp. 1–4.

[18] Lu X, Wang P, Niyato D, Kim DI, and Han Z, “Wireless networks with RF energy harvesting: A contemporary survey,” IEEE Commun. Surveys Tuts, vol. 17, no. 2, pp. 757–789, 2nd Quart., 2015.

[19] Papotto G, Carrara F, and Palmisano G, “A 90-nm CMOS threshold-compensated RF energy harvester,” IEEE J. Solid-State Circuits, vol. 46, no. 9, pp. 1985–1997, Sep. 2011.

[20] Chong G , “CMOS cross-coupled differential-drive rectifier in subthreshold operation for ambient RF energy harvesting—Model and analysis,” IEEE Trans. Circuits Syst. II, Exp. Briefs, vol. 66, no. 12, pp. 1942–1946, Dec. 2019.

[21] Saini G and Baghini MS, “A generic power management circuit for energy harvesters with shared components between the MPPT and regulator,” IEEE Trans. Very Large Scale Integr. (VLSI) Syst., vol. 27, no. 3, pp. 535–548, Mar. 2019.

[22] Shrivastava A, Roberts NE, Khan OU, Wentzloff DD, and Calhoun BH, “A 10 mV-input boost converter with inductor peak current control and zero detection for thermoelectric and solar energy harvesting with 220 mV cold-start and −14.5 dBm, 915 mHz RF kickstart,” IEEE J. Solid-State Circuits, vol. 50, no. 8, pp. 1820–1832, Aug. 2015.

[23] Chowdary G, Singh A, and Chatterjee S, “An 18 nA, 87% efficient solar, vibration and RF energy-harvesting power management system with a single shared inductor,” IEEE J. Solid-State Circuits, vol. 51, no. 10, pp. 2501–2513, Oct. 2016.

[24] Zargham M and Gulak PG, “A 0.13 μm CMOS integrated wireless power receiver for biomedical applications,” in Proc. ESSCIRC (ESSCIRC), 2013, pp. 137–140.

[25] Tavakol A and Staszewski RB, “An impedance sensor for MEMS adaptive antenna matching,” in Proc. IEEE Radio Freq. Integr. Circuits Symp. (RFIC), 2015, pp. 379–382.

[26] Chang TC, Weber M, Charthad J, Nikoozadeh A, Khuri-Yakub PT, and Arbabian A, “Design of high-efficiency miniaturized ultrasonic receivers for powering medical implants with reconfigurable power levels,” in Proc. IEEE Int. Ultrason. Symp. (IUS), 2015, pp. 1–4.

[27] Po FCW , “A 2.4GHz CMOS automatic matching network design for pacemaker applications,” in Proc. Joint IEEE North-East Workshop Circuits Syst. TAISA Conf, 2009, pp. 1–4.

[28] Khalifa A, Karimi Y, Huang Y, Stanaćevic M, and Etienne-Cummings R, “The challenges of designing an inductively coupled power link for μm-sized on-chip coils,” in Proc. IEEE Biomed. Circuits Syst. Conf. (BioCAS), 2018, pp. 1–4.

[29] Monti G , “EMC and EMI issues of WPT systems for wearable and implantable devices,” IEEE Electromagn. Compat. Mag, vol. 7, no. 1, pp. 67–77, 1st Quart., 2018.

[30] Kim C , “Design of miniaturized wireless power receivers for mm-sized implants,” in Proc. IEEE Custom Integr. Circuits Conf. (CICC), 2017, pp. 1–8.

[31] Moghaddam AK, Chuah JH, Ramiah H, Ahmadian J, Mak P-I, and Martins RP, “A 73.9%-efficiency CMOS rectifier using a lower dc feeding (LDCF) self-body-biasing technique for far-field RF energy-harvesting systems,” IEEE Trans. Circuits Syst. I, Reg. Papers, vol. 64, no. 4, pp. 992–1002, Apr. 2017.

[32] Abouzied MA, Ravichandran K, and Sánchez-Sinencio E, “A fully integrated reconfigurable self-startup RF energy-harvesting system with storage capability,” IEEE J. Solid-State Circuits, vol. 52, no. 3, pp. 704–719, Mar. 2017.

[33] Khalifa A , “The microbead: A highly miniaturized wirelessly powered implantable neural stimulating system,” IEEE Trans. Biomed. Circuits Syst, vol. 12, no. 3, pp. 521–531, Jun. 2018. 29877816 10.1109/TBCAS.2018.2802443

[34] Zeng Z , “Design of sub-gigahertz reconfigurable RF energy harvester from −22 to 4 dBm with 99.8% peak MPPT power efficiency,” IEEE J. Solid-State Circuits, vol. 54, no. 9, pp. 2601–2613, Sep. 2019.

[35] Xu P, Flandre D, and Bol D, “A self-gating RF energy harvester for wireless power transfer with high-PAPR incident waveform,” IEEE J. Solid-State Circuits, vol. 56, no. 6, pp. 1816–1826, Jun. 2021.

